# Role of *TCF7L2* risk variant and dietary fibre intake on incident type 2 diabetes

**DOI:** 10.1007/s00125-012-2634-x

**Published:** 2012-07-11

**Authors:** G. Hindy, E. Sonestedt, U. Ericson, X.-J. Jing, Y. Zhou, O. Hansson, E. Renström, E. Wirfält, M. Orho-Melander

**Affiliations:** 1 Department of Clinical Sciences in Malmö, Lund University, Clinical Research Center, Malmö, Sweden; 2 Diabetes and Cardiovascular Disease, Genetic Epidemiology, Department of Clinical Sciences, Lund University Diabetes Centre, Lund University, Clinical Research Centre 91:12, Jan Waldenströms gata 35, 20502 Malmö, Sweden

**Keywords:** Diet, Gene, Gene–environment interaction, Transcription factor 7-like 2 (TCF7L2), Type 2 diabetes

## Abstract

**Aims/hypothesis:**

The T allele of transcription factor 7-like 2 gene variant, *TCF7L2* rs7903146, increases the risk of type 2 diabetes by 40–50%. As *TCF7L2* rs7903146 has been associated with diminished incretin effect we investigated whether interaction between dietary intake of carbohydrate, fat, protein or fibre and this variant affects the risk of type 2 diabetes.

**Methods:**

A cohort of 24,799 non-diabetic individuals from the Malmö Diet and Cancer Study (MDCS), with dietary data obtained by a modified diet history method, were followed up for 12 years, with 1,649 recordings of incident type 2 diabetes made. Risk of type 2 diabetes in strata of diet quintiles was analysed prospectively adjusting for potential confounders. Cross-sectional analyses were performed on baseline fasting glucose and HbA_1c_ levels in a subset of 5,216 randomly selected individuals from the MDCS.

**Results:**

The elevated risk of type 2 diabetes with rs7903146 (OR 1.44, 95% CI 1.33, 1.56, *p* = 4.6 × 10^−19^) increased with higher intake of dietary fibre (OR 1.24, 95% CI 1.04, 1.47 to OR 1.56, 95% CI 1.31, 1.86 from the lowest to highest quintile; *p*_interaction_ = 0.049). High intake of dietary fibre was inversely associated with diabetes incidence only among CC genotype carriers (OR 0.74, 95% CI 0.58, 0.94 per quintile, *p* = 0.025). The T allele was associated with 0.027% elevated HbA_1c_ (*p* = 0.02) and this effect increased with higher intake of fibre (from −0.021% to 0.079% for the lowest to the highest quintile, *p*_interaction_ = 0.02). Each quintile of higher fibre intake was associated with lower HbA_1c_ levels among CC and CT but not among TT genotype carriers (−0.036%, *p* = 6.5 × 10^−7^; −0.023%, *p* = 0.009; and 0.012%, *p* = 0.52, respectively).

**Conclusions/interpretation:**

Our study suggests that dietary fibre intake may modify the association between *TCF7L2* rs7903146 and incidence of type 2 diabetes, and that higher fibre intake may associate with protection from type 2 diabetes only among non-risk allele carriers.

## Introduction

Transcription factor 7-like 2 gene (*TCF7L2*) rs7903146 to this date remains the strongest and most widely replicated type 2 diabetes susceptibility locus [1, 2]. In addition to the increased risk of type 2 diabetes, the *TCF7L2* rs7903146 T allele has been associated with increased fasting glucose and HbA_1c_ levels in genome-wide association studies (GWAS) [3, 4].

As a principal transcription factor in the wingless-type MMTV integration site (WNT) signalling pathway [5], TCF7L2 has been reported to be involved in the induction of transcription of the proglucagon gene through heterodimerisation with β-catenin and synthesis of glucagon-like peptide 1 (GLP-1) [6]. In line with this, several studies have reported an attenuated insulin response to oral glucose in individuals with the *TCF7L2* risk variant, pointing to the possibility of a defective incretin system [7, 8].

Levels of incretin hormones are modified by macronutrient intake [9, 10] and several previous studies have tested for interactions between *TCF7L2* risk variants and diet. In the Diabetes Prevention Program, the TT genotype of *TCF7L2* rs7903146 showed a tendency towards being more strongly associated with type 2 diabetes in the placebo group compared with the intervention group but the results did not reach statistical significance [11]. In the European Prospective Investigation into Cancer and Nutrition (EPIC) Potsdam cohort [12] higher whole-grain intake was found to be protective against type 2 diabetes among rs7903146 CC genotype carriers but not among T allele carriers. Still another study, a large meta-analysis of 14 cohorts, investigating fasting glucose levels instead of incident type 2 diabetes, did not detect any interaction between the *TCF7L2* risk allele and whole-grain intake on that phenotype [13]. In addition, the *TCF7L2* risk allele was reported to have a stronger association with type 2 diabetes among individuals with higher dietary glycaemic load and glycaemic index [14]. Finally, a recent report from the Tübingen Lifestyle Intervention Program (TULIP) described an interaction between dietary fibre and the *TCF7L2* rs7903146 risk variant with regard to successful weight loss after a lifestyle intervention [15].

In this study we hypothesised that different dietary intakes, in particular the relative intake levels of carbohydrates, fats, proteins or fibres, could modify the risk associated with the *TCF7L2* rs7903146 T allele in incident type 2 diabetes.

## Methods

### *Study population*

The Malmö Diet and Cancer Study (MDCS) is a population-based prospective cohort study based in the city of Malmö, Sweden. In 1991 the source population of the MDCS was defined to include all individuals born between 1926 and 1945 and living in Malmö. In 1995 this was extended to include all women born between 1923 and 1950 and men born between 1923 and 1945. This resulted in a source population of 74,138 individuals. Study participants were recruited through public advertisements or personal letters. Mental disability and limited Swedish language skills were used as the sole exclusion criteria. Study participants were invited to visit the screening centre twice during the baseline examination period, which extended from March 1991 to October 1996. During the first visit, participants were divided into groups of six to eight individuals and received instructions on how to record meals in the menu book. They were also instructed on how to fill in the diet questionnaire and the extensive questionnaire covering socioeconomic and lifestyle factors, to be completed at home. Approximately 10 days later, participants returned for a dietary history interview. By the end of the baseline examination period, we had complete dietary, anthropometric and lifestyle data on 28,098 individuals. Details of the recruitment procedures are described elsewhere [16].

From this population we excluded 909 individuals with prevalent type 2 diabetes, identified as individuals with a self-reported diabetes diagnosis or on a self-reported glucose-lowering regimen. After exclusion of prevalent type 2 diabetes patients we were left with 27,189 individuals, 24,799 of whom had available DNA samples and were genotyped successfully for *TCF7L2* rs7903146 and composed our study population. Of these, 15,010 were women (mean [±SD] age 57.3 ± 7.9 years, BMI 25.3 ± 4.2 kg/m^2^) and 9,789 were men (age 59.1 ± 3.4 years, BMI 26.2 ± 3.4 kg/m^2^).

Altogether 6,103 individuals were randomly selected from the MDCS to participate in a cardiovascular sub-cohort (MDC-CC). Additional measurements were obtained for these individuals, including analysis of fasting blood glucose and HbA_1c_ levels. For the analyses in the MDC-CC, we excluded cases of prevalent type 2 diabetes, and included 5,216 individuals with complete diet, fasting glucose and genotype information: 3,067 women (age 57.3 ± 5.9 years, BMI 25.3 ± 4.2 kg/m^2^) and 2,149 men (age 57.5 ± 6.0 years, BMI 26.1 ± 3.4 kg/m^2^).

The MDCS was approved by the Ethical Committee at Lund University (LU 51-90). All participants provided written informed consent.

### *Incident type 2 diabetes*

We studied the incidence of type 2 diabetes until December 2006 (mean follow-up time 11.8 ± 3.0 years). Incident cases were identified using the Swedish National Diabetes Register [17] and the Diabetes 2000 register of Skåne region [18]; both registers included only individuals diagnosed by a physician according to established guidelines. To identify cases that were not diagnosed at the hospital, we used the local HbA_1c_ register, which contains data from institutional and non-institutional care in Malmö since 1988 [19]. Individuals with at least two HbA_1c_ values above 6.0%, using the Swedish Mono-S standardisation system (corresponding to 6.9% using the US National Glycohemoglobin Standardization Program and 52 mmol/mol using International Federation of Clinical Chemistry and Laboratory Medicine units) [20, 21], were categorised as diabetes cases. In our study population (*n* = 24,799) a total of 1,649 incident cases of type 2 diabetes occurred during the follow-up period.

### *Dietary assessment*

An interview-based, modified dietary history method specially designed for the MDCS was used consisting of: (1) a 7-day menu book where lunch, dinner meals and cold beverages, including alcohol, were recorded; and (2) a dietary 168-item questionnaire to assess meal patterns, consumption frequencies and portion sizes of regularly consumed foods. Medicinal drugs, natural remedies and nutrient supplements were recorded in the menu book. A 48-page booklet was used to help participants at home estimate the portion sizes for recording information in the questionnaire. This was followed by interviews performed by trained interviewers. Portion sizes and dishes in the menu book were estimated during the interview using a more extensive book with photographs. Participants were also asked about their meal pattern, cooking methods and food choices.

Data from the menu book and diet questionnaire were used to calculate the average daily intake of foods. The average daily food intake was converted to energy and nutrient intakes using the Malmö Diet and Cancer Food and Nutrient Database, which was designed for the MDCS and was derived from PC KOST2-93 of the Swedish National Food Administration [22, 23].

A slight alteration of the coding routines for dietary data was introduced in September 1994 [23]. A method variable, classifying data collected before and after September 1994, along with a four-category season variable (i.e. winter, spring, summer and autumn) was created and used as a covariate to adjust for variation in data collection over time.

Dietary variables used in our analysis included total energy intake (EI) (kJ), carbohydrate, fat and protein intake as percentages of non-alcohol EI (%E), and fibre intake as grams (g) per 4,184 kJ (1,000 kcal). The relative validity of the dietary assessment method used in the MDCS has previously been evaluated in a sample of 50- to 69-year-old Malmö residents, 105 women and 101 men. The reference method used was 18 days’ weighed food records (3 days every second month) collected over 1 year. Energy-adjusted Pearson correlation coefficients for fat, carbohydrate, protein and fibre intake were in the range of 0.54–0.74 [24].

Individuals with potentially inaccurate reports of EI (*n* = 4,548) were identified as having a ratio of EI to the basal metabolic rate outside the 95% CI limits of the physical activity level (PAL) calculated for each individual as total energy expenditure. This procedure is described in detail elsewhere [25].

Individuals with a change in their dietary habits in the past (*n* = 5,540) due to illness or other factors were identified by one questionnaire item [22].

### *Other variables used as potential confounders*

Leisure-time physical activity was assessed by an extensive lifestyle questionnaire adapted from the Minnesota Leisure Time Physical Activity Questionnaire. Participants had to estimate the number of minutes per week for each season they spent performing each of 17 different physical activities. The duration was multiplied by an intensity factor to create a physical activity score that was divided into tertiles. Participants were classified as current smokers, ex-smokers and never-smokers. Alcohol intake was classified into four categories based on grams of alcohol consumed per day: zero, low (<15 g/day in women or <20 g/day in men), medium (15–30 g/day in women or 20–40 g/day in men) and high consumers (>30 g/day in women or >40 g/day in men). The education variable was created by classifying participants according to their highest educational level (≤8 years, 9–10 years and 11–13 years at school, and university degree).

### *Genotyping TCF7L2*

rs7903146 was genotyped using the TaqMan PCR method (Applied Biosystems, Foster City, CA, USA), according to the manufacturer's instructions. The ABI Prism Sequence Detection System ABI 7900HT (Applied Biosystems) was used for post-PCR allelic discrimination by measuring allele-specific fluorescence. The concordance rate was >99% in 325 randomly repeated samples. Genotyping success rate was 96%. The genotypes were in Hardy–Weinberg equilibrium (*p* = 0.16); 13,571 (54.7%) individuals carried the CC genotype, 9,488 (38.3%) the CT genotype and 1,740 (7.0%) the TT genotype.

### *Statistical analysis*

Assuming an additive model, logistic regression was used to calculate the OR of incident type 2 diabetes associated with the *TCF7L2* T risk allele in the MDCS, adjusting for age, sex and BMI. A similar analysis was done within quintiles of relative intakes of carbohydrate, fat, protein and fibre. Interactions between *TCF7L2* genotypes and quintiles of different dietary intakes and type 2 diabetes incidence were analysed by introducing a multiplicative factor of genotype and dietary quintiles as continuous variables and also adding these variables to the equation. Interactions were analysed using a basic adjustment model for age, sex, BMI, total EI, method and season. For the sensitivity analyses, we excluded inaccurate reporters of EI and in the prospective analysis of incident type 2 diabetes, we further excluded individuals reporting a change in their dietary habits.

In the MDC-CC subcohort we performed cross-sectional analysis using linear regression to calculate the effect sizes per each risk T allele on baseline fasting plasma glucose and HbA_1c_ in quintiles of fibre intake, adjusting for age, sex and BMI. Interactions between quintiles of dietary fibre intake and *TCF7L2* genotype on fasting plasma glucose and HbA_1c_ were analysed by introducing a multiplicative factor of genotype and dietary quintiles as continuous variables using the same adjustment model as described for the MDCS above. For the sensitivity analyses, we excluded individuals potentially reporting inaccurate EI.

QUANTO (http://hydra.usc.edu/gxe/ accessed 1 March 2012) was used to calculate the statistical power for the gene–diet interaction with incident type 2 diabetes and baseline HbA_1c_ levels [26, 27]. Assuming an OR of 0.90 per fibre quintile (additive model) and an OR of 1.44 per *TCF7L2* T allele (26% allele frequency, additive model) on type 2 diabetes incidence, and an effect of −0.028% per fibre quintile and 0.027% per *TCF7L2* T allele on HbA_1c_ levels, we had 80% power to detect an interaction OR of at least 1.08 in type 2 diabetes incidence, and an interaction effect of at least 0.022% on HbA_1c_ levels.

Our analyses showed similar results after further adjustments for potential confounders as physical activity, alcohol intake, smoking habits and level of education.

We used IBM SPSS Statistics, version 19 (SPSS Inc., Chicago, IL, USA), for the analyses. Two-sided *p* values of <0.05 were considered significant.

## Results

The *TCF7L2* rs7903146 T allele was associated with a 44% (95% CI 33, 56) increased risk of incident type 2 diabetes (*p* = 4.6 × 10^−19^) in the MDCS. In the MDC-CC subcohort, each additional rs7903146 T allele was associated with 0.059 mmol/l higher fasting plasma glucose (*p* = 0.004) and 0.027% (0.27 mmol/mol) higher HbA_1c_ (*p* = 0.02) level (Table 1). Different genotype carriers reported similar mean intakes of total energy, carbohydrates, fats, protein and fibre (Table 1).

**Table 1:** Characteristics of the MDCS cohort by *TCF7L2* genotype

Characteristic	*TCF7L2* genotype			OR (95% CI)^a^ or β (SE)^b^	*p*_trend_^c^
	CC	CT	TT		
*n*	13,571	9,488	1,740		
Incident T2DM (%)	741 (5.5)	757 (8.0)	151 (8.7)	1.44 (1.33, 1.56)^a^	4.6 × 10^−19^
FPG (mmol/l)^d^	5.6 ± 0.9	5.7 ± 0.9	5.7 ± 0.8	0.06 (0.02)	0.004
FPI (pmol/l)^d^	46.8 ± 43.6	47.6 ± 52.6	44.1 ± 27.2	0.22 (1.00)	0.83
HbA_1c_ (%)^d^	4.8 ± 0.5	4.8 ± 0.5	4.9 ± 0.5	0.03 (0.01)	0.02
HbA_1c_ (mmol/mol)^d^	39.7 ± 5.2	40.0 ± 5.3	40.1 ± 4.8	0.27 (0.12)	0.02
Age (years)	58.1 ± 7.6	58.0 ± 7.6	58.0 ± 7.6	−0.08 (0.07)	0.30
BMI (kg/m^2^)	25.7 ± 3.9	25.7 ± 3.9	25.5 ± 3.8	−0.08 (0.04)	0.06
Energy (kJ)	9,548 ± 2,728	9,535 ± 2,711	9,569 ± 2,874	5.06 (24.2)	0.28
Carbohydrate (%E)	45.2 ± 6.0	45.2 ± 6.1	45.2 ± 5.9	0.01 (0.06)	0.99
Fat (%E)	39.1 ± 6.1	39.0 ± 6.2	39.1 ± 6.0	−0.02 (0.06)	0.75
Protein (%E)	15.7 ± 2.6	15.8 ± 2.5	15.8 ± 2.5	0.01 (0.03)	0.79
Fibre (g/4,184 kJ)	9.0 ± 2.7	9.0 ± 2.7	9.1 ± 2.8	0.003 (0.025)	0.91

Data are means ± SD unless otherwise stated

No. of individuals included in MDCS cohort, *n* = 24,799

^a^Logistic regression model assuming an additive genetic model adjusting for age, sex and BMI

^b^β represents the difference generated by each additional T allele

^c^General linear model, assuming an additive genetic model adjusting for age, sex, BMI, season and method where appropriate

^d^Data available only for the MDC-CC, *n* = 5,216

FPG, fasting plasma glucose; FPI, fasting plasma insulin; T2DM, type 2 diabetes mellitus

No significant interactions were found between rs7903146 and quintiles of carbohydrate, fat or protein intake (*p* = 0.91, 0.47 and 0.70, respectively) and incident type 2 diabetes (Table 2). However, the risk of type 2 diabetes with the *TCF7L2* T allele increased from 24% to 56% from the lowest (mean intake: 5.8 ± 0.8 g/4,184 kJ) to the highest (mean intake: 13.1 ± 2.2 g/4,184 kJ) quintile of fibre intake (*p*_interaction_ = 0.049). In the sensitivity analysis excluding potential inaccurate reporters of EI (18.3% of the study sample), the interaction between rs7903146 and quintiles of fibre intake was more evident (*p* = 0.006) (Table 2). The interaction remained significant after further exclusion of individuals who reported a dietary change in the past (resulting in exclusion of 35.9% of the study sample) (*p* = 0.046).

**Table 2:** OR of incident type 2 diabetes by *TCF7L2* rs7903146 genotype and quintiles of different dietary intakes in the MDCS

Dietary component	Mean intake	OR (95% CI)^a^				*p*_trend_^a^	*p*_interaction_^a,b^
		CC	CT	TT	Additive model		
Carbohydrate (%E)							0.91 (0.44)
Q1	36.9	1.00 (ref)	1.54 (1.21, 1.95)	2.09 (1.40, 3.11)	1.49 (1.25, 1.77)	8.3 × 10^−6^	
Q2	42.1	1.00 (0.79, 1.26)	1.36 (1.07, 1.73)	1.62 (1.05, 2.51)	1.32 (1.10, 1.58)	2.9 × 10^−3^	
Q3	45.2	0.92 (0.72, 1.17)	1.62 (1.28, 2.05)	1.85 (1.24, 2.76)	1.53 (1.29, 1.82)	9.5 × 10^−7^	
Q4	48.2	0.87 (0.68, 1.11)	1.37 (1.07, 1.76)	1.71 (1.12, 2.60)	1.47 (1.23, 1.76)	3.0 × 10^−5^	
Q5	53.7	0.91 (0.71, 1.16)	1.47 (1.16, 1.88)	1.34 (0.83, 2.17)	1.37 (1.14, 1.64)	9.0 × 10^−4^	
*p*_trend_		0.30	0.74	0.19			
Fat (%E)							0.47 (0.17)
Q1	30.5	1.00 (ref)	1.55 (1.23, 1.96)	1.63 (1.06, 2.51)	1.38 (1.16, 1.64)	3.0 × 10^−4^	
Q2	35.9	0.73 (0.57, 0.94)	1.29 (1.01, 1.66)	2.03 (1.36, 3.03)	1.69 (1.41, 2.03)	1.9 × 10^−8^	
Q3	39.0	0.98 (0.77, 1.24)	1.47 (1.15, 1.86)	1.55 (1.03, 2.35)	1.36 (1.14, 1.61)	5.8 × 10^−4^	
Q4	42.2	0.88 (0.69, 1.12)	1.45 (1.14, 1.85)	1.53 (0.99, 2.38)	1.45 (1.21, 1.73)	5.1 × 10^−5^	
Q5	47.6	0.98 (0.77, 1.24)	1.37 (1.07, 1.75)	1.66 (1.07, 2.58)	1.36 (1.13, 1.64)	9.4 × 10^−4^	
*p*_trend_		0.64	0.61	0.55			
Protein (%E)							0.70 (0.52)
Q1	12.5	1.00 (ref)	1.57 (1.21, 2.05)	1.81 (1.15, 2.86)	1.44 (1.19, 1.74)	2.2 × 10^−4^	
Q2	14.4	1.12 (0.87, 1.45)	1.87 (1.45, 2.41)	1.69 (1.06, 2.69)	1.40 (1.17, 1.68)	2.3 × 10^−4^	
Q3	15.6	1.02 (0.79, 1.32)	1.65 (1.27, 2.13)	2.02 (1.29, 3.15)	1.49 (1.24, 1.80)	3.0 × 10^−5^	
Q4	16.9	1.25 (0.97, 1.60)	1.66 (1.28, 2.14)	2.18 (1.42, 3.33)	1.33 (1.12, 1.59)	1.5 × 10^−3^	
Q5	19.4	1.24 (0.97, 1.60)	2.09 (1.62, 2.68)	2.63(1.78, 3.89)	1.51 (1.29, 1.77)	3.5 × 10^−7^	
*p*_trend_		0.12	0.10	0.04			
Fibre (g/4,184 kJ)							0.049 (0.006)
Q1	5.8	1.00 (ref)	1.35 (1.08, 1.70)	1.33 (0.86, 2.05)	1.24 (1.04, 1.47)	1.4 × 10^−2^	
Q2	7.5	0.74 (0.58, 0.93)	1.09 (0.86, 1.38)	1.39 (0.90, 2.16)	1.43 (1.18, 1.72)	2.0 × 10^−4^	
Q3	8.7	0.80 (0.63, 1.01)	1.29 (1.03, 1.63)	1.70 (1.15, 2.50)	1.52 (1.28, 1.80)	1.8 × 10^−6^	
Q4	10.1	0.73 (0.57, 0.93)	1.14 (0.90, 1.46)	1.48 (0.98, 2.26)	1.49 (1.24, 1.80)	2.1 × 10^−5^	
Q5	13.1	0.74 (0.58, 0.94)	1.41 (1.11, 1.79)	1.44 (0.94, 2.22)	1.56 (1.31, 1.86)	8.3 × 10^−7^	
*p*_trend_		0.029	0.78	0.64			

No. of individuals included in MDCS cohort, *n* = 24,799

^a^Basic model with adjustments for age, sex, BMI, total EI, season and method

^b^Sensitivity analysis after excluding inaccurate reporters of EI using the basic model

CC, CT, TT denotes *TCF7L2* genotype; ref denotes reference value

**Fig. 1:**
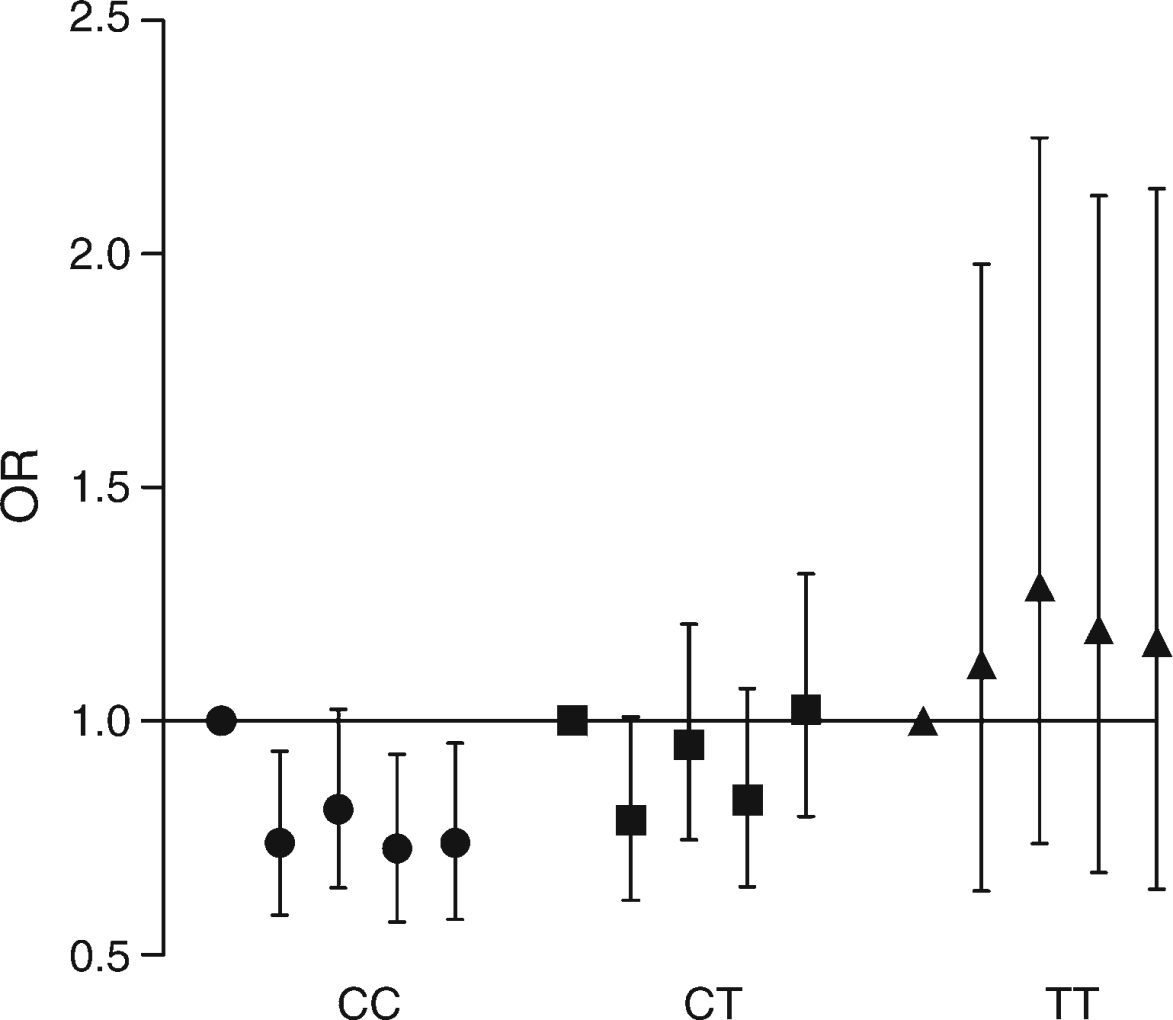
ORs of type 2 diabetes in quintiles of fibre intake in strata of *TCF7L2* genotype in MDCS (*n* = 24,799). We used the first quintile as a reference (OR 1) and adjusted for age, sex, BMI, total EI, season and method. Comparing the highest and lowest quintiles, a higher fibre intake was only protective among CC (circle) genotype carriers (*p*_trend_ = 0.025). Higher fibre intakes were not associated with type 2 incidence among CT (square) (*p*_trend_ = 0.77) and TT (triangle) (*p*_trend_ = 0.60) genotype carriers. The error bars denote the 95% CI

Since in several earlier studies fibre intake has been associated with protection against type 2 diabetes, we next analysed the effect of fibre intake on the risk of type 2 diabetes among different *TCF7L2* genotype carriers. When comparing the extreme groups of fibre intake (i.e. the highest quintile vs the lowest) separately within each genotype group, we found that higher fibre intake was associated with protection against type 2 diabetes among CC genotype carriers (OR 0.74, 95% CI 0.58, 0.94, *p*_trend_ = 0.025), but not among CT or TT genotype carriers (CT: OR 1.03, 95% CI 0.80, 1.32, *p*_trend_ = 0.77; TT: OR 1.13, 95% CI 0.62, 2.07, *p*_trend_ = 0.60) (Fig. 1).

**Fig. 2:**
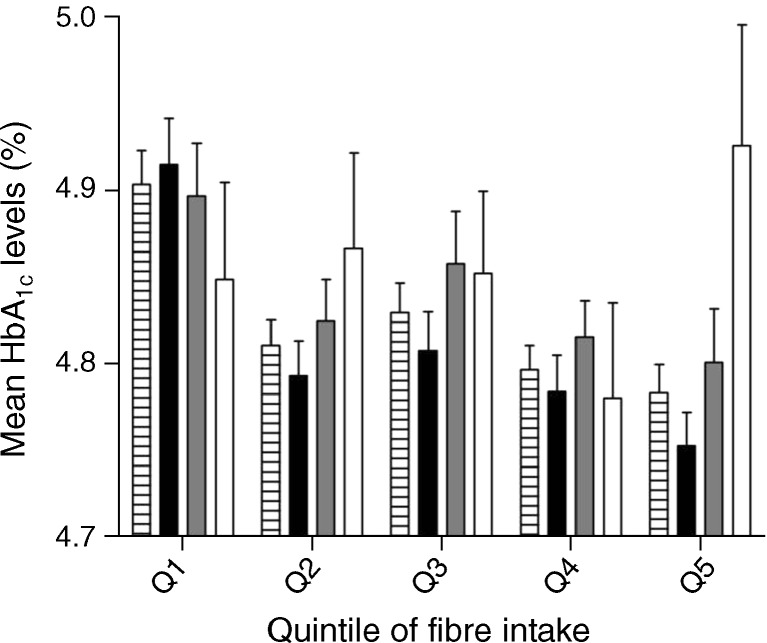
Mean HbA_1c_ levels in quintiles of fibre intake, by the *TCF7L2* rs7903146 genotypes, in the MDC-CC (*n* = 5,216). The associated effect size (β) per T allele on HbA_1c_ level increased in higher fibre intake groups (*p*_interaction_ = 0.020) and the risk allele was associated with higher HbA_1c_ levels only in the highest quintile of fibre intake (β = 0.08%, *p* = 0.002). Individuals in the highest quintile of fibre intake had significantly lower HbA_1c_ levels compared with those in the lowest intake group (*p* < 0.001). This association was driven by the strong associated effect per fibre intake quintile among the CC genotype carriers (β = −0.036%, *p* = 6.5 × 10^−7^). No such association was observed among TT genotype carriers (β = 0.012, *p* = 0.52) while carriers of both alleles appeared as an intermediate group (β = −0.023%, *p* = 0.009). To convert values for HbA_1c_ in % into mmol/mol, multiply by 10.11 and subtract 8.94. Genotype carriers: hatched bar, all; black bar, CC; grey bar, CT; white bar, TT. The error bars denote the SEM

We next performed cross-sectional interaction analyses of the quantitative traits of fasting glucose and HbA_1c_ that have been reported to be associated with the *TCF7L2* variant in GWAS (Table 3). In the MDC-CC we did not detect any significant interaction between quintiles of fibre intake and *TCF7L2* genotype and baseline fasting plasma glucose levels (*p* = 0.20). However, the association with elevated baseline HbA_1c_ levels increased significantly with higher fibre intake (effect size −0.021% (−0.21 mmol/mol) to 0.079% (0.80 mmol/mol) per T allele from the lowest to highest quintile, *p*_interaction_ = 0.020), and the T allele was significantly associated with higher HbA_1c_ levels only in the highest quintile of fibre intake (*p*_trend_ = 0.002) (Fig. 2). This result remained significant in a sensitivity analysis after excluding inaccurate reporters of EI (*p*_interaction_ = 0.033).

**Table 3:** Mean fasting plasma glucose and HbA_1c_ by quintiles of fibre intake and *TCF7L2* genotype

Quintiles	*TCF7L2* genotype			Effect size^a^	*p*_trend_^a^	*p*_*interaction*_^b^
	CC	CT	TT			
Mean fasting glucose (mmol/l)						
Fibre (g/4,184 kJ)						
Q1	5.85	5.84	5.73	−0.006	0.91	0.20
Q2	5.65	5.70	5.83	0.09	0.03	
Q3	5.60	5.69	5.74	0.10	0.01	
Q4	5.59	5.60	5.67	0.009	0.80	
Q5	5.53	5.63	5.71	0.12	0.006	
Effect size^b^	−0.04	−0.03	−0.03			
*p*_trend_^b^	0.0002	0.047	0.39			
Mean HbA_1c_ % (mmol/mol)						
Fibre (g/4,184 kJ)						
Q1	4.92 (40.8)	4.90 (40.6)	4.85 (40.1)	−0.021 (−0.21)	0.49	0.02
Q2	4.79 (39.5)	4.82 (39.4)	4.87 (40.3)	0.032 (0.33)	0.16	
Q3	4.81 (39.7)	4.86 (40.2)	4.85 (40.1)	0.033 (0.33)	0.21	
Q4	4.78 (39.4)	4.82 (39.7)	4.78 (39.4)	0.011 (0.12)	0.60	
Q5	4.75 (39.1)	4.80 (39.6)	4.93 (40.9)	0.079 (0.80)	0.002	
Effect size^b^	−0.036 (−0.37)	−0.023 (−0.24)	0.012 (0.13)			
*p*_trend_^b^	6.5 × 10^−7^	0.009	0.52			

Data are taken from *n* = 5,216 individuals

^a^Basic model with adjustments for age, sex and BMI

^b^Basic model with adjustments for age, sex, BMI, total EI, season and method

We then analysed the association between fibre intake and HbA_1c_ levels among different *TCF7L2* genotype carriers (Table 3). Among all MDC-CC individuals, higher fibre intake was associated with lower HbA_1c_ levels (−0.03% [−0.3 mmol/mol], −0.04% [−0.4 mmol/mol] to −0.02% [−0.2 mmol/mol] per quintile of fibre intake, *p*_trend_ = 1.7 × 10^−4^). This protective association was strongest among CC genotype carriers (−0.036% [−0.37 mmol/mol] per quintile, *p* = 6.5 × 10^−7^), while a higher fibre intake was not associated with HbA_1c_ levels among TT genotype carriers (0.012% [0.13 mmol/mol], *p* = 0.52) and carriers of both alleles appeared as an intermediate group (−0.023% [−0.24 mmol/mol], *p* = 0.009) (Fig. 2).

As *TCF7L2* rs7903146 has been, in several studies, shown to associate more strongly with risk of type 2 diabetes among lean as compared with overweight individuals, we were concerned that some potential confounding could still be present after adjusting for BMI. In the MDCS the risk of type 2 diabetes associated with the *TCF7L2* T allele decreased from 86% to 31% from the lowest to highest BMI quintile (*p*_interaction_ = 0.009). However, dietary fibre intake and BMI did not correlate (*r* ^2^ = −0.006, *p* = 0.88) and separate interaction analyses of BMI tertiles indicated similar results in each BMI category.

Finally, each quintile of higher leisure-time PAL was associated with 6.4% reduced risk of type 2 diabetes (*p* = 0.0003). However, PAL did not interact with *TCF7L2* genotype on type 2 diabetes incidence (*p* = 0.46), and the interaction between fibre intake and *TCF7L2* genotype on type 2 diabetes incidence remained similar after further adjustment for leisure-time physical activity.

## Discussion

Although type 2 diabetes is thought to result from a complex interplay between genetic predisposition and an unfavourable environment, very little is known about the interactions involved. We observed the risk increase of type 2 diabetes with the rs7903146 T allele to be significantly accentuated by increasing dietary fibre intake. Analyses of HbA_1c_ levels supported this observation as the rs7903146 T allele was only associated with higher HbA_1c_ levels among individuals with the highest fibre intake.

Several previous studies have reported a protective association between a high fibre intake and type 2 diabetes [28, 29]. Our results indicate that the protective effect of higher fibre intake is dependent on the genetic background of the individual, being limited to *TCF7L2* non-risk CC genotype carriers. This is in line with a recent report from the prospective EPIC-Potsdam case-control study reporting that the association between whole-grain intake and protection against type 2 diabetes is dependent on *TCF7L2* rs7903146 genotype; a high whole-grain intake was associated with protection among CC genotype carriers while individuals carrying one or two T alleles lacked such protection [12]. Our analyses of HbA_1c_ levels by *TCF7L2* genotype and fibre intake further support such an interaction, as the *TCF7L2* T allele was associated with higher HbA_1c_ levels only among individuals with higher fibre intake. Consistent with the observed dissimilar effects of fibre intake on type 2 diabetes incidence among different *TCF7L2* genotype carriers, a high fibre intake was strongly associated with lower HbA_1c_ levels among CC genotype carriers, while this association was completely lacking among TT genotype carriers. We did not find any interaction between dietary fibre intake and *TCF7L2* variant on fasting glucose levels, which is in line with a large meta-analysis of 14 cohorts (including MDC-CC), which reported no interaction between whole-grain intake and *TCF7L2* variant and fasting glucose levels [13].

The major strengths of our study include the high relative validity of our dietary assessment method, the combination of a diet diary with a questionnaire, the large sample size, the prospective design and the ability to identify inaccurate reporters of energy intake and individuals who had changed their diet in the past. In addition, the obtained association between higher dietary fibre intake and lower risk of type 2 diabetes and HbA_1c_ levels suggests that the dietary and type 2 diabetes incidence measures of the MDCS are adequate. Still, our study suffers from limitations including projection of the baseline diet data to the whole follow-up period in the prospective analyses (type 2 diabetes) and the limited causal inference in the cross-sectional analyses (HbA_1c_ levels). In addition, we did not correct the statistical analyses for multiple comparisons as the dietary variables are correlated and we had the possibility of repeating the test of interaction between *TCF7L2* rs7903146 and fibre intake on HbA_1c_ levels. Despite these limitations our interaction data from the prospective analyses were supported by the data obtained using a cross-sectional design. However, we need to keep in mind that the observed significance levels of the interactions were not robust and thus the possibility of false-positive findings cannot be excluded and therefore our results need to be replicated in other studies.

In our study, the protective association of higher dietary fibre intake with type 2 diabetes incidence was restricted to around 55% of the population who were non-carriers of the *TCF7L2* risk allele, while TT genotype carriers completely lacked such protection and CT carriers appeared as an intermediate group. Fibre intake has been associated with lower postprandial glucose and insulin concentrations, which have been mainly attributed to slower intestinal absorption of nutrients [30]. In our study this was reflected among the CC genotype carriers who had a significantly lower HbA_1c_ level as well as a significantly lower incidence rate of type 2 diabetes when reporting high fibre intake. Dietary fibre has, in previous studies, been associated with inconsistently affected GLP-1 response, which could be due to differences in studied fibre types, the limited number of individuals studied and/or the short duration of the studies [31, 32]. However, it has been shown in hyperinsulinaemic individuals that after 9–12 months, higher fibre intake was associated with elevated plasma short-chain fatty acids (SCFAs), which are products of colonic fermentation of dietary fibre, and higher GLP-1 levels [33], pointing to a long-term effect of dietary fibre on glucose homeostasis. Several animal studies have shown that SCFAs are associated with increased expression of the proglucagon gene and GLP-1 secretion in rat intestinal cells [34–36]. At least part of the protective association of dietary fibre with the risk of type 2 diabetes could therefore be mediated by SCFAs through increased GLP-1 release. Since the *TCF7L2* T allele has previously been associated with an impaired incretin effect [7], it can be speculated that carriers of this risk allele could suffer from some degree of incretin resistance, leading to a lack of benefit from higher GLP-1 levels associated with SCFAs from higher fibre intake. This could be of clinical relevance, especially as many type 2 diabetes patients are on incretin-based treatment regimens and the risk allele carriers may benefit from these drugs to a lesser extent.

However, as systemic plasma SCFAs have previously been reported to increase after fermentable dietary fibre intake [37, 38], SCFAs may affect other tissues, such as pancreatic islets. Among the different SCFAs, butyrate has been identified as the most potent histone deacetylase inhibitor (HDACi) [39], which may be of interest because the rs7903146 risk variant sequence has been reported to confer an islet-specific open chromatin state translating to an elevated enhancer effect on *TCF7L2* transcription [40]. Butyrate as a fermentation product of dietary fibre could therefore play a role in further propagating the previously reported difference between the rs7903146 T allele carriers and the non-carriers via histone hyperacetylation, which may result in further enhanced transcription of the already overexpressed risk transcript. Another possibility could be the ability of an HDACi to increase the levels of active β-catenin [41].

To conclude, our study suggests that the *TCF7L2* risk variant modifies the protective association of dietary fibre intake with type 2 diabetes incidence and HbA_1c_ levels. Although our epidemiological observations cannot be translated into dietary advice for carriers of the *TCF7L2* risk allele, our results question whether a fibre-rich diet is protective against type 2 diabetes in all individuals, and by which mechanisms such protection may be lost in T allele carriers. Further studies are needed to answer these questions and to understand the mechanisms by which the *TCF7L2* risk variant increases the risk of type 2 diabetes.

## Abbreviations

%E: Percentage of non-alcohol energy intake
EI: Energy intake
EPIC: European Prospective Investigation into Cancer and Nutrition
GLP-1: Glucagon-like peptide 1
GWAS: Genome-wide association studies
HDACi: Histone deacetylase inhibitor
MDC-CC: Malmö Diet and Cancer Study, cardiovascular cohort
MDCS: Malmö Diet and Cancer Study
PAL: Physical activity level
SCFA: Short-chain fatty acid
TCF7L2: Transcription factor 7-like 2
